# Implementing the Practice of Birth Companion in Labor During the COVID-19 Pandemic: A Quality Improvement Endeavor in India

**DOI:** 10.7759/cureus.30531

**Published:** 2022-10-20

**Authors:** Latika Chawla, Madhulika Singh, K Aparna Sharma, Shalini Rajaram, Amrita Gaurav, Kavita Khoiwal, Rajlaxmi Mundhra, Anupama Bahadur, Ria M, Nevetha Ravichandran, Jaya Chaturvedi

**Affiliations:** 1 Obstetrics and Gynecology, All India Institute of Medical Sciences, Rishikesh, Rishikesh, IND; 2 Obstetrics and Gynecology, All India Institute of Medical Sciences, New Delhi, New Delhi, IND

**Keywords:** covid-19, pareto principle, respectful maternity care (rmc), quality improvement (qi), birth companion (bc)

## Abstract

Objective

We aim to implement the practice of birth companions (BC) (from 0% to 90%) during labor to provide respectful maternity care (RMC) during the coronavirus disease 2019 (COVID-19) pandemic.

Methods

This was a prospective quality improvement (QI) study conducted in the Department of Obstetrics and Gynecology at All India Institute of Medical Sciences (AIIMS), Rishikesh, India. The methodology given by the World Health Organization (WHO)’s Point of Care Continuous Quality Improvement (POCQI) manual was followed, and standard tools of quality improvement were used to attain the objective.

Results

The QI team conducted a cause and effect analysis to understand the reasons why birth companions were not allowed during childbirth. The Pareto principle derived at three most important causes of the problem: absence of a defined policy, ignorance of guidelines promoting BC even during the pandemic, and relatives could enter wards only after a negative reverse transcriptase polymerase chain reaction (RTPCR) report, which could take up to 48 hours. Multiple change ideas were tested by means of Plan-Do-Study-Act (PDSA) cycles that were successful in bringing about desired change and improvement in the delivery of quality healthcare.

Conclusion

QI methodology was effective in promoting and achieving more than 90% birth companionship in labor and thus helpful in providing respectful maternity care even during the COVID-19 pandemic.

## Introduction

The World Health Organization (WHO) defines respectful maternity care (RMC) as “care organized for and provided to all women in a manner that maintains their dignity, privacy, and confidentiality, ensures freedom from harm, mistreatment, and enables informed choice and continuous support during labor and childbirth” [1]. RMC is the fundamental right of every woman, and disrespectful and abusive behavior during labor and childbirth is a violation of this fundamental right [2]. However, it is a widely accepted fact that women very often suffer abuse, disrespect, and neglect during labor and childbirth in health facilities across the world, especially in developing nations [3]. Women’s interactions with healthcare providers can either empower and comfort them or cause long-term psychological and emotional trauma, boosting or lowering their confidence and self-esteem [3].

The coronavirus disease 2019 (COVID-19) pandemic has succeeded in shaking humanity, challenging the delivery of optimum healthcare to the needy while ensuring the safety of healthcare workers (HCWs). Sadly, pregnant women were one of the most vulnerable groups of people adversely affected worldwide. Unfortunately, expectant mothers have been frequently on the receiving end of undignified behavior at the hands of healthcare workers. The modified work atmosphere because of this pandemic just added fuel to the fire. There have been significant reports stating that the COVID-19 pandemic has negatively affected the ability of healthcare workers to provide respectful maternity care globally [4].

Restriction of access of family members/attendants to their laboring patient in the wards and difficulty in the identification of healthcare workers (HCWs) designated for this specialized care due to the use of personal protective equipment (PPE) were some of the reasons for the lack of RMC these pregnant women rightfully deserved.

Respectful maternity care is a broad concept with a birth companion (BC) at its core. It is but natural that the presence of the husband/a relative/family member/friend or a doula during the process of birth and thereafter will not just allay birth-related anxiety, decrease stress, and provide confidence to the birthing woman but also mitigate possible abusive behavior (physical and verbal) from the various cadres of HCWs [5]. The WHO recommends that all women should have access to a birth companion of their choice [2]. Birth companions are commonly allowed in private maternity facilities in the country; however, very few government setups in India allow birth companions [6].

Our center was designated as a tertiary care COVID-19 facility in the state of Uttarakhand, India. At our own facility, which is a government tertiary care COVID-19 center, we realized that we are not able to meet the standards of RMC due to the transformed working conditions and changes. This motivated us to allow birth companions in an attempt to provide RMC during the COVID-19 pandemic. We started this endeavor in September 2020, when the first wave of the pandemic peaked in India.

The Ministry of Health and Family Welfare (MoHFW), Government of India, started the Labor Room Quality Improvement Initiative (LaQshya) in 2017 [7]. This initiative has been targeted to improve the delivery of intrapartum and postpartum care at healthcare facilities. The guideline has a patient-centric approach and focuses on the improvement in quality of care and interaction (attitude, behavior, and language) between patients and healthcare workers with an aim to provide respectful maternity care in government health-providing facilities [7]. According to this initiative, 90% of deliveries in any setup should be accompanied by a birth companion. These guidelines were our basis for forming the objective of our quality improvement (QI) project [7].

## Materials and methods

Objective

We aim to establish the practice of allowing birth companions (BC) during the process of birth in the labor room of our institute from 0% to 90% during the ongoing COVID-19 pandemic.

Methods

This was a prospective quality improvement study conducted at the All India Institute of Medical Sciences (AIIMS), Rishikesh, India. The study was approved by the institutional ethical committee (AIIMS/IEC/20/501) on August 8, 2020. Patients were enrolled according to the inclusion and exclusion criteria. Antenatal patients undergoing vaginal delivery at the institute who wish/chose to have a birth companion and patients without any ongoing intrapartum or postpartum complications were included in the study. This included COVID-19-negative and COVID-19 suspected cases. Antenatal patients who had an instrumental vaginal delivery or a cesarean section who did not want to have a birth companion and patients who had a known COVID-19-positive status were excluded from the study. Informed written consent was taken from patients.

Quality improvement (QI) methodology as given by the WHO’s Point of Care Continuous Quality Improvement (POCQI) manual was followed, and standard tools of quality improvement were applied as explained below [8].

Team formation

For this QI improvement, a team was constituted consisting of the head of the department, a faculty member, a resident from each unit of the Department of Obstetrics and Gynecology, a nursing in-charge of the department, and two nursing officers posted in the labor room. The corresponding author acted as the team leader. Baseline data over two weeks was collected. This data revealed clearly that in our institute, birth companions were not allowed during the process of childbirth.

Problem statement

At the start of the pandemic, the obstetric facility was divided into two discrete areas in two different parts of the hospital: a COVID-19-positive facility and a COVID-19-negative facility. The COVID-19-positive facility had a holding area for COVID-19 suspects and another area for known COVID-19-positive patients. The COVID-19-negative obstetric facility was an area in another part of the hospital. All elective patients were advised to come for admission with a COVID-19 report. Patients who came with COVID-19-positive reports were directly taken in the COVID-19-positive area. Patients who came with COVID-19-negative reports were taken in the COVID-19-negative area. All patients reaching the obstetric emergency without a COVID-19 report were considered to be suspects. Emergency patients with symptoms of COVID-19 infection were segregated from the asymptomatic suspects. All areas had negative pressure ventilation, and beds were at least 3 m apart. Unfortunately, the patient load was high, and we did not have enough isolation rooms to accommodate all. Informed written consent was taken from all patients prior to admission. COVID-19-appropriate behavior was followed by all. They were shifted to either the COVID-19-positive or COVID-19-negative wards only after the reverse transcriptase polymerase chain reaction (RTPCR) report. Due to the significant workload in the institute, the turnover time for an RTPCR report was anywhere from 24 to 48 hours, and antenatal/laboring women were shifted to the COVID-19-negative obstetric ward/labor room only after the results of patients were RTPCR-negative. All healthcare workers in the COVID-19-positive and suspect areas worked wearing full PPE.

Cause and effect analysis (fishbone diagram)

**Figure 1:**
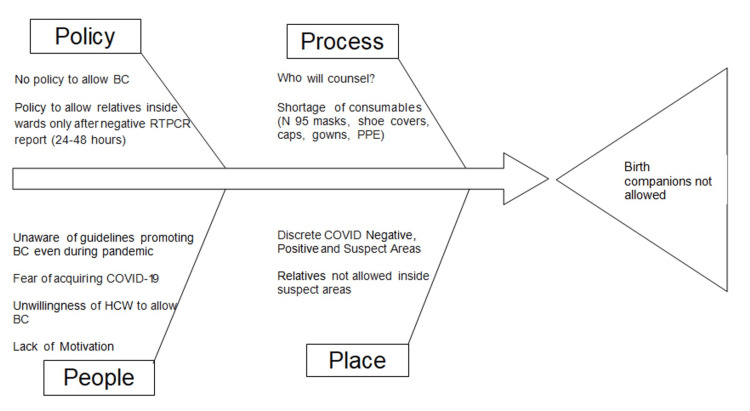
Fishbone diagram (cause and effect diagram) of the reasons why birth companions were not allowed during childbirth BC: birth companion, RTPCR: reverse transcriptase polymerase chain reaction, PPE: personal protective equipment, COVID-19: coronavirus disease 2019, HCW: healthcare worker

The QI team conducted a cause and effect analysis (fishbone diagram) to understand the reasons why birth companions were not allowed during childbirth. A total of 45 healthcare workers were interviewed (nursing officers and residents) to find out the reasons why birth companions were not allowed during labor in our facility. Discussions were held with residents and staff nurses posted in both COVID-19-positive and COVID-19-negative obstetric facilities. The problems identified after discussions were categorized under four subheadings: people, process, place, and policy (Figure 1).

Application of the Pareto principle

**Figure 2:**
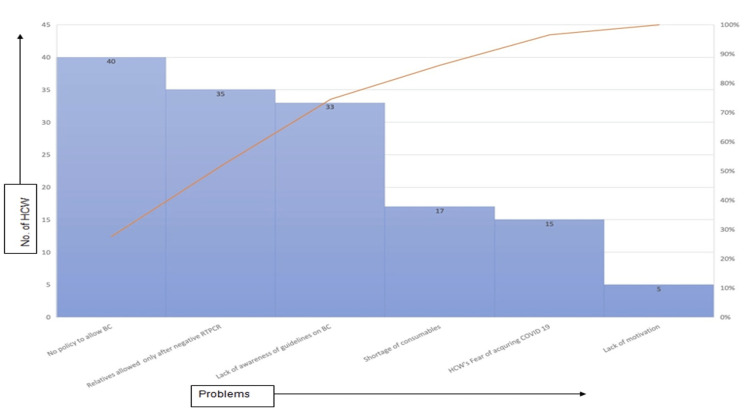
Pareto chart showing the identification of the most important causes leading to our problem BC: birth companion, COVID-19: coronavirus disease 2019, HCW: healthcare worker, No.: number, RTPCR: reverse transcriptase polymerase chain reaction

The application of the Pareto principle helped us identify 20% of the causes that lead to 80% of the problem. The absence of a defined policy, ignorance of guidelines promoting BC even during the pandemic, and the fact that relatives could enter wards only after a negative RTPCR report, which could take up to 48 hours, were the top three reasons leading to the majority of the problem (Figure 2).

Maternal care flow diagram

**Figure 3:**
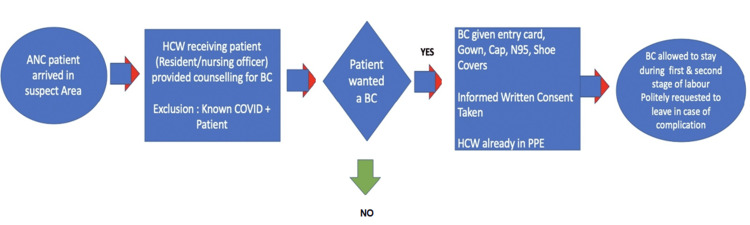
Maternal care flow diagram ANC: antenatal care, HCW: healthcare worker, BC: birth companion, COVID: coronavirus disease, N95: N95 masks, PPE: personal protective equipment

A maternal care flow diagram was constructed to enable the smooth implementation of our plan of allowing birth companions during delivery in both COVID-19 suspect and COVID-19-negative facilities simultaneously. Healthcare workers receiving the patient at admission were assigned the responsibility of counseling regarding the availability of the option of a birth companion. The senior resident on duty was given the overall responsibility of supervising this process. If the patient chose to have a birth companion, informed written consent (about the risks of acquiring COVID-19 infection from the hospital premises and ward) was obtained, and the birthing partners were issued an entry card. They were provided with protective gear including gowns, caps, shoe covers, and N95 masks. It was imperative for duty teams working in COVID-19 suspect areas to be donned in full PPE for their own protection. The birth companion was allowed to stay during the first and second stages of labor. In case of any complication during the second stage, they were politely requested to leave the area (Figure 3).

## Results

Our quality indicator was the percentage of eligible women who had a birth companion with them during delivery, and the target value was decided at 90% according to the national guidelines [7]. Baseline data collected in the month of September showed that birth companions were not allowed in even a single delivery so far (0%).

The team discussed and planned multiple interventions targeting the most important causes by way of Plan-Do-Study-Act (PDSA) cycles.

PDSA 1: Online orientation class for HCW regarding guidelines and the importance of BC

**Figure 4:**
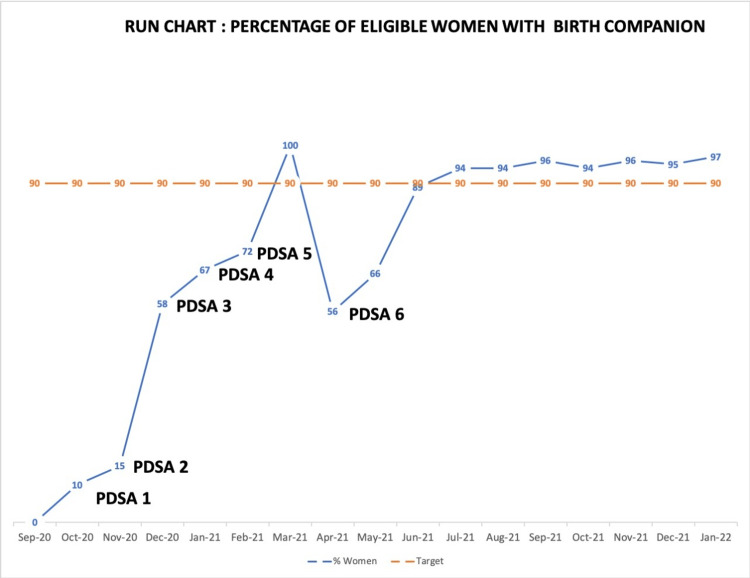
Run chart depicting quality indicator over time as birth companion rate improved and reached target level after six PDSA cycles PDSA: Plan-Do-Study-Act

An online class was taken for the residents and nursing officers where the basics of respectful maternity care were introduced to them. The importance of birth companionship was emphasized. They were familiarized with the national Indian guidelines (LaQshya) [7], Indian Council of Medical Research (ICMR) [9] guidelines, and international guidelines such as WHO [3] and Royal College of Obstetricians and Gynaecologists (RCOG) [10], which emphasizes on the importance of a birthing partner to allow a birth companion even during the COVID-19 pandemic albeit with proper screening and protective gear. The birth companion rate reached 10% after the first intervention (Figure 4).

PDSA 2: Policy

BC was made mandatory for all deliveries (exclusion: known COVID-19-positive patients and patients who refuse to have a BC). 

A departmental policy of having a birth companion in each delivery was formed. All members of the department were intimated of the same. Exclusions included known COVID-19-positive patients, patients who refuse to have a BC, non-availability of BC, or any obstetrical complication that happened during the second stage of labor that required the companion to leave the delivery room.

Companions were allowed in the COVID-19 suspect areas (even without an RTPCR report) after informed written consent and after providing them with protective gear. The birth companion rate improved to 15% after the second intervention (Figure 4).

PDSA 3: Formation of a WhatsApp group

**Figure 5:**
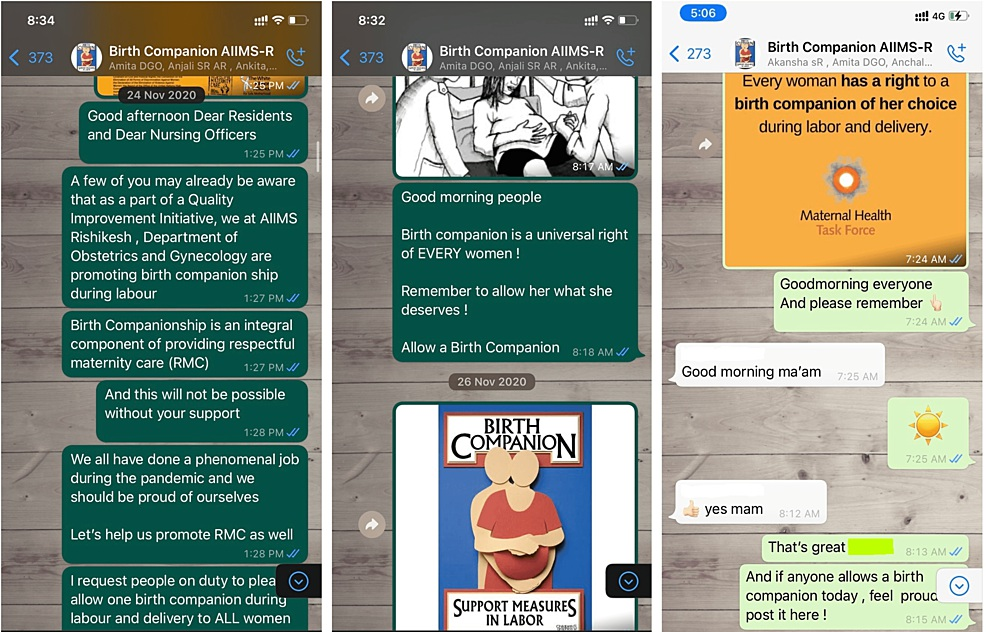
Snapshots from the WhatsApp group that was created as a PDSA intervention PDSA: Plan-Do-Study-Act

A WhatsApp group named “Birth Companion AIIMS-R” was formed, and residents and nursing officers from the department were added to the same. Frequent reminders and posters about the importance of birth companions were posted. Residents were encouraged to take a picture during childbirth (with the consent of the patient and companion, maintaining their dignity and modesty) and share it with the group. There was positive reinforcement by applauding those allowing BC (teams that were found to be allowing birth companions) in the group with each delivery (Figure 5). The birth companion rate reached 58% after this intervention (Figure 4).

PDSA 4: Department members who were not allowing BC during their duties

Those who were not allowing BC were identified. Discussions were held with them in person, and their problems were heard out. Of the patients, 67% were now accompanied by birth companions after this intervention (Figure 4).

PDSA 5: Shortage of consumables

Issues were discussed with the medical superintendent’s office, respective officers in the hospital store were intimated, and continuous supply was thus ensured. The birth companion rate reached 72% after the fifth intervention and reached 100% within six months of starting our study (Figure 4).

PDSA 6: A steep fall in our quality indicator in the seventh month of the endeavor

The birth companion rate reached 56% (Figure 4). This happened because a new batch of residents who were unaware of the concept of birth companionship joined the team. They were oriented to our exercise and were added to the WhatsApp group. The indicator improved to 89% in two months.

**Figure 6:**
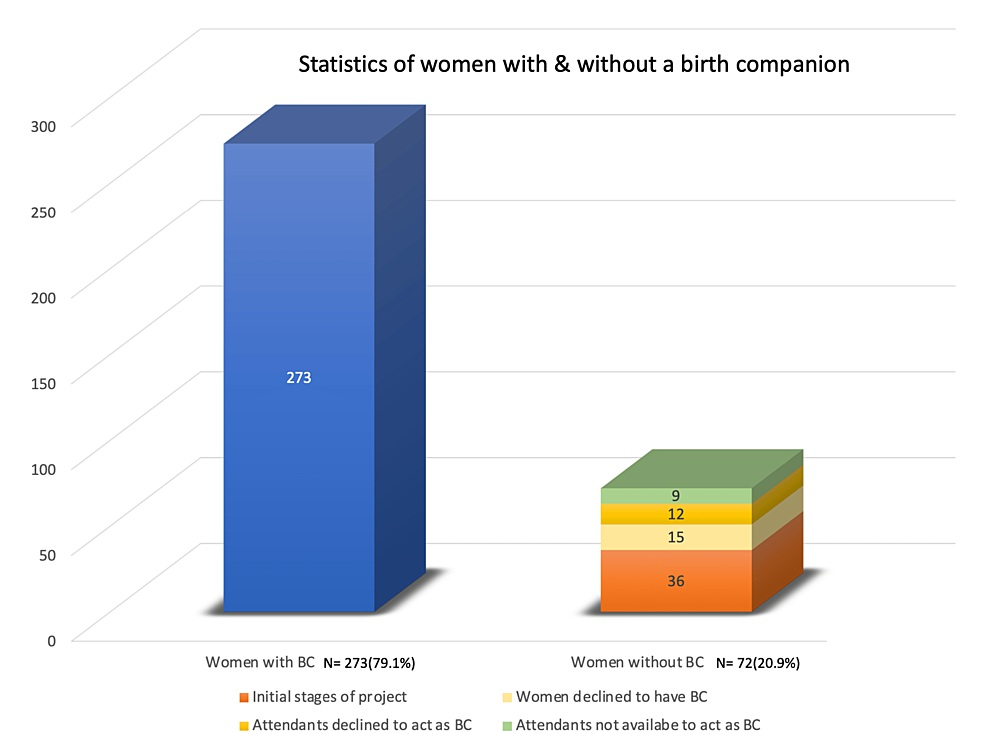
Statistics of women with and without a birth companion BC: birth companion

A total of 345 women were included in the study, out of which 273 (79.1%) were accompanied by a birth companion. Of them, 72 (20.9%) could not be accompanied by a birth companion; the reasons for this are shown in Figure 6. Thirty-six (10.4%) patients could not have a birth companion as we did not have a policy for the same and we were in the initial stages of the implementation of our quality improvement endeavor. Fifteen (4.3%) patients declined to have a birth companion. The attendants of 12 (3.4%) patients refused to act as birth companions. The attendants of nine (2.6%) patients were not available to act as birth companions as they were COVID-19-positive.

Sustainability

From the ninth month onward, we have been sustaining our quality indicator values above the target levels (Figure 4). A session was carried out (in the 12th month from the start of the endeavor) to congratulate the department members on the success of our study and to sustain the achievement of our quality target indicator. A PowerPoint presentation (Microsoft Corp., Redmond, WA, USA) was used to re-emphasize to the HCWs the importance of respectful maternity care and birth companions along with the national and international guidelines [3,9,10]. In the future, we plan to implement an introductory session on RMC and BC for each fresh batch of residents of our department that will help further sustain this improvement in care.

## Discussion

The International Childbirth Initiative states that every woman and newborn, irrespective of their background, color, caste, education, and socioeconomic status, deserve to have the best standards of healthcare and maternity services. Respectful maternity care is a fundamental right of every woman, and this also includes consideration and compassion even in the event of any complications [5]. A recent Cochrane review concluded that continuous support by a birth companion during labor may better outcomes for mother and neonates, including a greater percentage of vaginal birth, decreased cesarean section and instrumental delivery rates, shorter duration of labor, use of labor analgesia, better five-minute Apgar scores, and less negative feelings about childbirth [11]. Disrespectful behavior includes unsupportive activities such as not sharing information, not involving the patient in decision-making, verbal assaults, and physically harming women such as beating, hitting, slapping, and rough handling or even denial of care and abandonment [12,13]. Abusive and discourteous behavior in health facilities may not just lead to adverse maternal and newborn outcomes but may also prevent the mother from seeking future care in the same/any other healthcare facility. A recent cross-sectional Indian study attempted to explore the status of birth companionship in various tertiary care health facilities in the country. They found that despite awareness of the importance and advantages of birth companions, it was the absence of defined hospital policies, lack of space, lack of privacy, and crowding in labor rooms that were reported as reasons birth companions were not allowed. They also reported that the presence of birth companions was negatively associated with incidences of disrespect and abuse of women [6]. Childbirth is a very personal event in a woman’s life, and the experiences (positive or negative) stay with her for a very long time. Abusive behavior in health facilities can lead to a lifetime of mental and emotional scarring, humiliation, and regret. Therefore, the WHO strongly supports and propagates respectful maternity care [1,3,10].

Healthcare workers faced multiple challenges working during the pandemic, and these times were a first for most of them. A few of the many challenges we dealt with include the fear of acquiring the infection and then carrying it home to our family members. Working long shifts sweating in personal protective equipment (PPE), without eating/drinking water, was extremely difficult. Going from one ward to another required repeated donning and doffing of the PPE kits, which was extremely cumbersome and frustrating. The loud noise of the negative pressure fans made communication extremely difficult. Patients and their families struggled alike to cope with the changed systems. If there was anything more demoralizing and disheartening than being sick, it was being sick and dying without having kin by your side to provide you comfort, support, love, and care. The COVID-19 pandemic succeeded in doing just that. Patients died alone in the facilities. Relatives could not be around to provide support and care and say their goodbyes. Pregnant women had never been more vulnerable and in need of support. We aimed to provide just that with this intervention.

A similar quality improvement study was conducted at a tertiary care center in North India in 2019 [14]. They established a birth companion rate from 0% to 50% in all eligible women within six weeks. This study was however conducted prior to the pandemic. We took six months to achieve a 100% rate.

As a tertiary care center, our own restrictions to provide RMC motivated us to initiate this project. Also, this important intervention of allowing BC during childbirth seemed feasible without requiring extra logistics or resources that are difficult to obtain in a government setup like ours. Resistance was faced from orthodox faculty members of the institute initially; however, with continuous perseverance, they could be convinced to let us start with the study.

Strengths of the study

**Figure 7:**
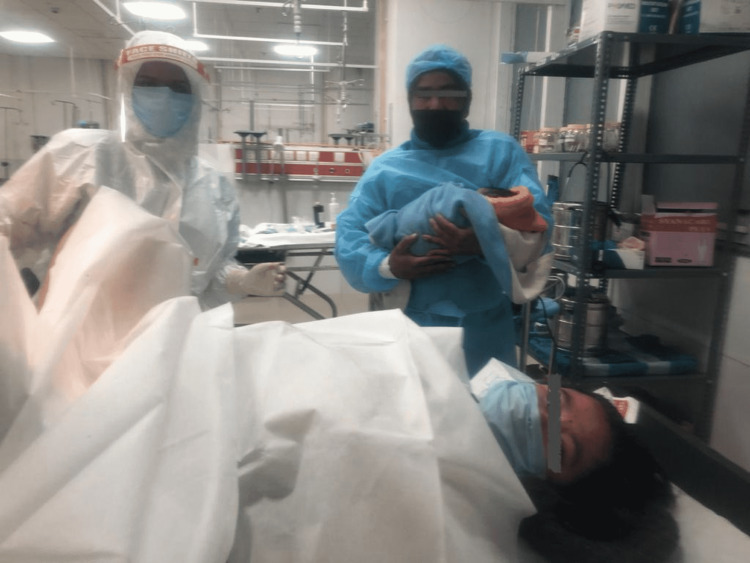
Implementing BC: birthing partner allowed in the COVID-19 suspect labor room BC: birth companion, COVID-19: coronavirus disease 2019

In a center without an existing policy to allow birth companions, the COVID-19 pandemic motivated us to initiate and establish the same. The team effort has been pivotal to our success. Positive reinforcement kept members of the department motivated to carry on with the quality work. The study utilized well-accepted tools for quality improvement. The fishbone analysis diagram helped us identify all the causes, and we were able to pick out the most important causes using the Pareto principle that helped us form and implement the multiple targeted change ideas in the form of PDSA cycles. PDSA cycles were successful in bringing about desired change and improvement in the delivery of quality healthcare (Figure 7).

Limitations of the study

We have not yet been able to include birth companions for women undergoing cesarean sections. We plan to do this intervention in the second leg of our endeavor.

## Conclusions

The simple steps of the WHO’s quality improvement (QI) methodology were effective in achieving our objective of attaining more than 90% rate of birth companions during labor and childbirth and promoting respectful maternity care even during the COVID-19 pandemic at our institute. It helped us achieve and sustain the national target figure as given by the Labor Room Quality Improvement Initiative (LaQshya), National Health Mission, Ministry of Health and Family Welfare (MoHFW), Government of India. We are maintaining our target indicator even now that the pandemic is over.
